# Editorial: Zebrafish: bona fide pathophysiological models of human diseases

**DOI:** 10.3389/fcell.2025.1624614

**Published:** 2025-06-27

**Authors:** Nicola Facchinello, Antionette L. Williams, Natascia Tiso

**Affiliations:** ^1^ Departments of Pharmacy and Biotechnology, University of Bologna, Bologna, Italy; ^2^ IRCCS Istituto delle Scienze Neurologiche di Bologna, Programma di Neurogenetica, Bologna, Italy; ^3^ Neuroscience Institute, Italian Research Council (CNR), Padova, Italy; ^4^ Division of Ophthalmology, Ann and Robert H. Lurie Children’s Hospital of Chicago, Chicago, IL, United States; ^5^ Department of Ophthalmology, Northwestern University Feinberg School of Medicine, Chicago, IL, United States; ^6^ Department of Biology, University of Padova, Padua, Italy

**Keywords:** zebrafish, *danio rerio*, model, mutant, human disease

**FIGURE 1:**
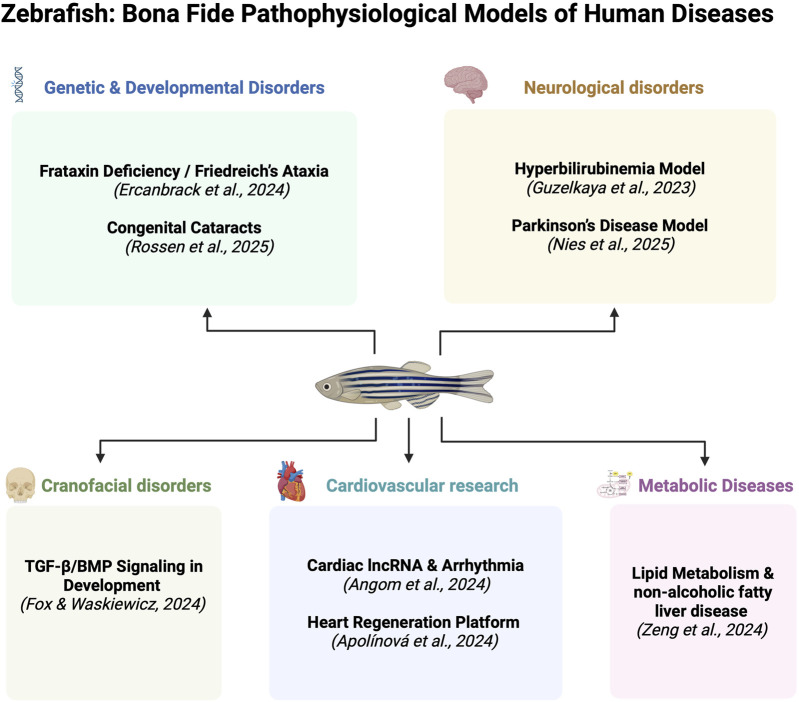
Zebrafish as Bona Fide Pathophysiological Models of Human Diseases. The figure summarizes examples of human diseases modeled in zebrafish. The references correspond to the contributions included in the current Research Topic as discussed in this Editorial. The figure was created with BioRender.com.

The zebrafish (*Danio rerio*) has emerged as a pivotal vertebrate model in biomedical research, occupying a unique position at the intersection of developmental biology, disease modeling, and translational medicine. With remarkable genetic conservation, optical transparency, rapid embryonic development, and a rich repertoire of genetic tools, zebrafish are now extensively employed to unravel the molecular and cellular mechanisms underlying a broad range of human diseases. This Research Topic gathers a compelling set of eight studies that illustrate the versatility and power of zebrafish in modeling human pathophysiology across diverse organ systems (Figure 1).

Neurodevelopmental and neurodegenerative diseases are well represented in this Research Topic. Guzelkaya et al. present a zebrafish model of neonatal hyperbilirubinemia to investigate bilirubin-induced neurological dysfunction (BIND), a condition still poorly understood in human neonates. Their study shows that bilirubin exposure impairs zebrafish locomotion and brain morphology, providing a vertebrate platform for dissecting bilirubin toxicity and testing neuroprotective interventions (Guzelkaya et al.).

In a related exploration of nervous system pathology, Nies et al. employ a rotenone-induced Parkinson’s disease model in zebrafish to assess the therapeutic potential of human metallothionein II (hMT2). Their findings reveal that hMT2 not only mitigates dopaminergic neurodegeneration and oxidative stress but also restores locomotor function, highlighting zebrafish as a translational platform for screening neuroprotective compounds (Nies et al.).

The vertebrate cardiac system is another focal point addressed in this Research Topic. Angom et al. use a forward genetic screen with gene-breaking trap lines to uncover *grin2bbART*, a novel long non-coding RNA that regulates calcium handling in the zebrafish heart. This study underscores the capacity of zebrafish as a model for uncovering regulators of heart physiology that might otherwise remain elusive in mammalian systems (Angom et al.). In a related study, Apolínová et al. present *ZebraReg*, an innovative automated platform that leverages the innate ability of zebrafish to regenerate heart tissue after injury. This tool allows for high-throughput screening of genes and compounds that influence cardiac regeneration, with clear translational implications for regenerative medicine in humans (Angom et al.).

In the domain of metabolic disease, Zeng et al. investigate *cobll1a*, a gene encoding a putative cytoskeletal protein involved in lipid metabolism. Their work shows that *cobll1a*-deficient zebrafish exhibit hepatic lipid accumulation due to disrupted retinoic acid signaling, linking this pathway to metabolic disorders, such as fatty liver disease and obesity. These findings reinforce the relevance of zebrafish for modeling and delineating the pathophysiological origins of complex metabolic syndromes (Zeng et al.).

Zebrafish also continue to provide crucial insights into developmental genetics and organogenesis. Ercanbrack et al. study the effects of *frataxin* knockdown in zebrafish embryos, modeling Friedreich’s Ataxia—a neurodegenerative disorder with mitochondrial dysfunction. These authors report defects in pronephros (early kidney) formation and embryonic development, expanding our understanding of the systemic roles of *frataxin* and its links to renal phenotypes in human disease (Ercanbrack et al.).

Zebrafish are immensely useful and popular genetic models for studying craniofacial development. In the present Research Topic, Fox and Waskiewicz review the contributions of zebrafish to understanding the roles of TGF-β and BMP signaling pathways in craniofacial morphogenesis. These authors highlight how zebrafish models replicate key features of congenital anomalies, such as cleft palate and craniosynostosis, offering a tractable system to study gene-environment interactions that influence facial structure (Fox and Waskiewicz).

Finally, Rossen et al. offer a comprehensive review of how zebrafish are used to model congenital cataracts, particularly those involving crystallin gene mutations. Given the high degree of conservation in lens development between zebrafish and humans, these models allow for real-time imaging of lens transparency and the testing of gene variants implicated in pediatric vision loss. This study highlights the zebrafish as a valuable model for both basic ocular research and therapeutic screening (Rossen et al.).

Together, these contributions exemplify the breadth and depth of zebrafish disease modeling. Across these studies, a variety of methodological approaches are employed, including CRISPR-based gene editing, morpholino knockdowns, transgenic reporters, forward genetic screens, and high-throughput imaging and behavioral assays. These tools enable fine-scale dissection of cellular and molecular processes *in vivo* and at scale—an asset particularly valuable for preclinical discovery pipelines.

Importantly, several studies in this Research Topic employ advancements beyond modeling toward therapeutic exploration. Nies et al. demonstrate the efficacy of hMT2 in ameliorating Parkinsonian phenotypes (Nies et al.), while *ZebraReg* offers a pipeline for regenerative compound discovery (Apolínová et al.). Such efforts underscore the translational potential of zebrafish not just as disease models but also as platforms for therapeutic innovation.

This Research Topic of studies also reflects the ability of zebrafish modeling to integrate systems-level insights. For example, links between developmental signaling and metabolic disease (Zeng et al.), or mitochondrial dysfunction and renal development (Ercanbrack et al.), illustrate the capacity of zebrafish models to reveal inter-organ and cross-pathway relationships that mirror human physiology and pathology.

Altogether, these studies affirm the zebrafish as a *bona fide* model of human disease, revealing the application of these animals in elucidating disease mechanisms, uncovering new gene functions, and screening therapeutics for a multitude of diseases ranging from monogenic conditions to complex multifactorial disorders. As research advances toward more patient-specific and systems-based approaches, zebrafish remain a cornerstone of translational research, bridging molecular discoveries to practical applications for the prevention of diseases and improvement of human health outcomes.

